# Optimizing Color Performance of the Ngenuity 3-Dimensional Visualization System

**DOI:** 10.1016/j.xops.2021.100054

**Published:** 2021-08-24

**Authors:** Samuel A. Minaker, Ryan H. Mason, David R. Chow

**Affiliations:** 1 Department of Ophthalmology, St. Michael’s Hospital, Toronto, Canada; 2 Department of Ophthalmology & Vision Sciences, University of Toronto, Toronto, Canada

**Keywords:** Color accuracy, Medical imaging, Ngenuity, 3D visualization, White balance, AFX, air-fluid exchange, HDR, high dynamic range, OLED, organic light-emitting diode, 3D, 3-dimensional, sRGB, standard red-green-blue

## Abstract

**Purpose:**

To evaluate the effect of surgeon-controlled parameters on the color performance of the Ngenuity 3-dimensional (3D) visualization system.

**Design:**

A calibrated reference target was placed inside a model eye to assess the Ngenuity 3D camera under different settings. The Ngenuity 3D display was assessed with a commercial colorimeter.

**Methods:**

Manufacturer-recommended methodology for white balancing was compared against all common deviations in technique. Following white balance, images of a calibrated reference target were extracted and tested using Imatest Master software to calculate quantitative color differences (delta E and delta C). The Ngenuity monitor was assessed using a SpyderX Elite commercial colorimeter to assess for image burn-in by quantifying color uniformity and maximum luminescence.

**Main Outcome Measures:**

Delta E and delta C were calculated for all variables. Color uniformity and luminance were assessed in candelas per square meter (nits).

**Results:**

Color performance using the manufacturer-recommended specifications yielded a delta E of 12.81 ± 1.67. Changing the white balance target to a videography grey card (*P* = 0.07) and 4 × 4 gauze (*P* = 0.37) provided similar performance, whereas using white computer paper or the operator’s palm significantly increased the delta E from 12.81 ± 1.67 to 15.28 ± 1.22 (*P* = 0.01) and 17.71 ± 2.03 (*P* < 0.01), respectively. Changes to card position, magnification, stability, or ambient lighting did not significantly impact white balance results, whereas having the card in crisp focus did decrease color accuracy (15.78 ± 1.63; *P* = 0.03). Minor improvement in performance occurred when the laser filter was off for white balance and image acquisition (9.28 ± 0.25; *P* < 0.01), but deterioration occurred if the laser filter was placed after balancing (16.59 ± 1.17; *P* < 0.01). Both light sources of 23-gauge light pipe at 34% intensity and 25-gauge chandelier at 50% intensity gave similar color accuracy (*P* = 0.37). When comparing different Ngenuity machines, color uniformity and maximum luminescence decreased with increased device use.

**Conclusions:**

Overall, the Ngenuity 3D has robust color performance. A few limitations of both the camera and monitor were identified, and surgeons should be aware of these pitfalls as well as solutions examined herein to mitigate their effects during surgery.

The Ngenuity system (Alcon Laboratories) was the first platform available for digitally assisted visualization systems. It has been adopted increasingly for its ability to improve ergonomics and depth of field, to filter digitally, to provide a high dynamic range, to integrate both endoscopy and intraoperative OCT, and to decrease phototoxicity.^1^ Many of these benefits are made possible by its use of a high dynamic range (HDR) camera and digital display.

Although digital camera sensors have improved over the past decade, they have inherent limitations that require meticulous white balance and adequate illumination for accurate color performance.^2^^,^^3^ In addition to the HDR camera, the organic light-emitting diode (OLED) monitor contributes to the color performance. However, image retention or burn-in is a known limitation of OLED displays.^4^ An OLED is a self-emissive technology where each pixel generates its own light, and over time, these pixels gradually dim. Pixels can degrade in a nonuniform manner when static images or graphics are displayed for prolonged periods.

Although these concerns are theoretical, inaccurate color performance with Ngenuity has been shown to occur in operating room settings (Supplemental Fig S1), and currently, little is known regarding parameters that surgeons can control readily to mitigate such inaccuracies. Despite these concerns, quantifying the color accuracy of this system has not been attempted.

The International Commission on Illumination was established in the early 1900s to standardize fields within color science, including image capture and display. In 1976, it defined a color space called CIE-L∗A∗B, where L represents luminance, A represents color on a green-red scale, and B represents color on a blue-yellow scale (Fig 1A).^5^ Therefore, color can be mapped and represented in 3-dimensional (3D) space. The distance between 2 colors in this 3D space is termed delta E and allows for the systematic quantification of color differences that previously could be described only with adjectives. A delta E value of less than 1 is not perceptible by the human eye, and a value of 100 is the exact opposite color. Typical delta E values are shown in Table 1.

**Figure 1:**
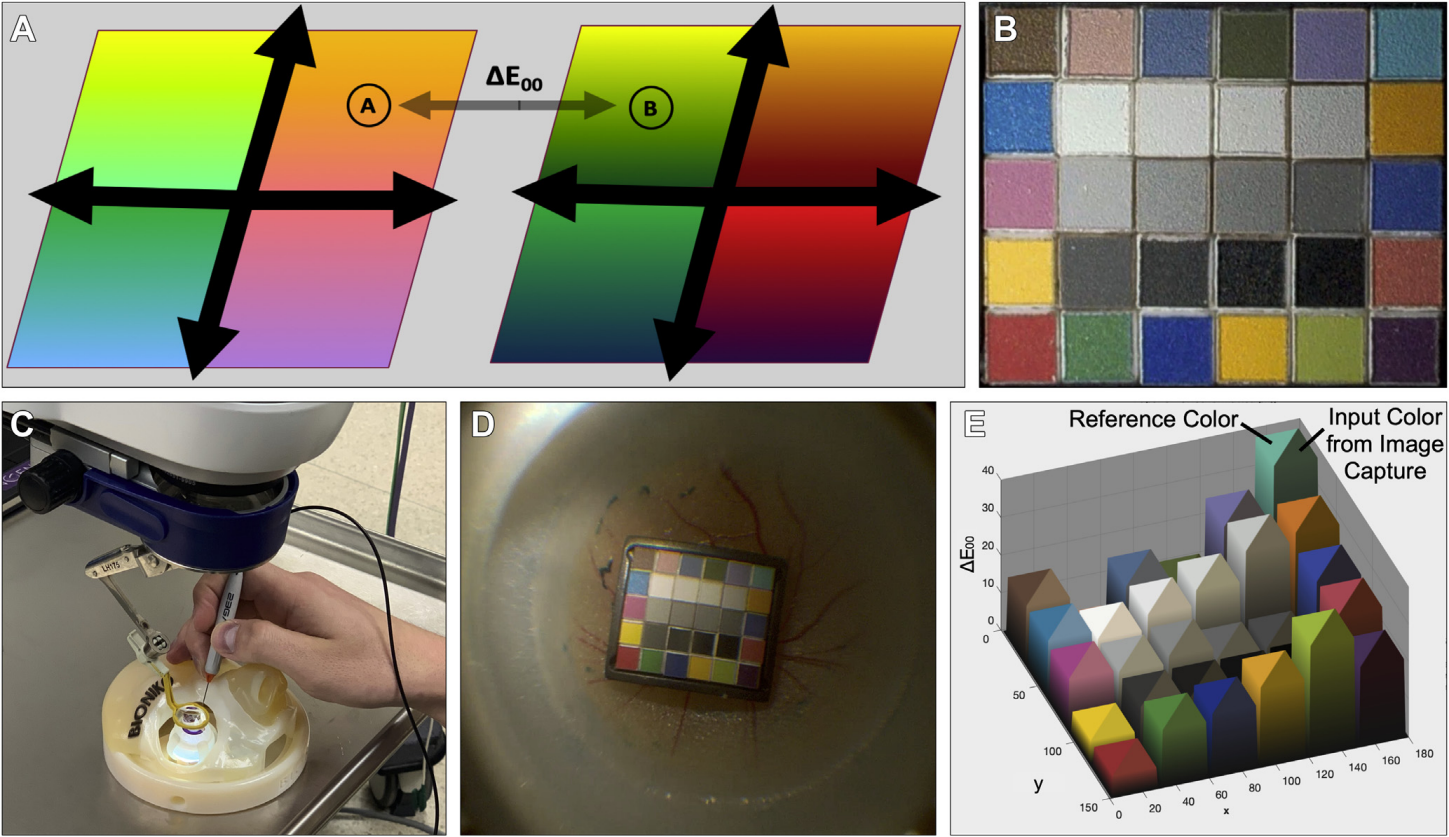
Experimental setup for color analysis: (**A**) delta E calculation factoring differences in chroma, hue, lightness, and saturation in a standardized color space to quantify perceived color differences; (**B**) ColorGauge 6 × 5 target; (**C**) model eye with light pipe illumination source; (**D**) photograph of the color target inside the model eye; and (**E**) example of data output from the Imatest Master analysis software.

**Table 1:** Summary of Delta E Values with Their Associated Level of Human Perception

ΔE_00_	Perception
< 1.0	Not perceptible by human eyes
1–2	Perceptible through close observation
3–10	Perceptible at a glance
11–49	Colors are more similar than opposite
100	Colors are exact opposites

Herein, we describe a novel methodology and apply it to quantify the color accuracy and performance of the Ngenuity system while assessing both the HDR camera and the OLED monitor. To our knowledge, this is the first study to assess the color performance of the Ngenuity 3D visualization system.

## Methods

### Experimental Design

Detailed methodology can be found in Supplement File S1. The imaging target used in color assessment was the ISA ColorGauge 6 × 5 grid (Image Science Associates; Fig 1B), designed for color, tone, and full-scale white balance of cameras and scanners in close view and macrophotography applications. Colors within this chart match the GretagMacbeth target that has been an industry standard since 1976. Colors were chosen to represent natural objects, colors that are problematic for color representation, additive and subjective primaries, and a grey scale. The color target was placed into a Vitreoretinal Fundus model of the eye (Bioniko). The same color target was used for all experiments and was handled based on manufacturer recommendations. Individual patient-level consent was not required. All research adhered to the tenets of the Declaration of Helsinki.

Experiments were completed in 3 operating room settings across 2 sites, the first at St. Michael’s Hospital, University of Toronto, Toronto, Canada (machine 1) and the second and third systems at the Kensington Eye Institute, Toronto, Canada (machines 2 and 3). Unless otherwise indicated, room lights were turned off before experimentation. The illuminance in the operating room was measured using the Light Spectrum Pro application with an Apple iPhone XS model as this has been shown to have an accuracy within 2% to 8% of professional devices and is readily user accessible. The ambient room light was measured to be 14 lux at St. Michael’s Hospital and 6 lux at the Kensington Eye Institute.

The Ngenuity systems at both sites used a Zeiss Lumera 700 (Carl Zeiss Meditec) operating microscope equipped with a ×1.75 condensing lens and a passive 532-nm laser filter in place for all experiments unless otherwise indicated. Ngenuity software version 1.2 was used under the Xenon setting, which has the same color lookup profile as the Xenon and Constellation setting in software update versions 1.3 and 1.4 to allow our data to be more generalizable. Unless otherwise indicated, data were acquired under the Posterior imaging mode. A digital, high dynamic range, 3D camera was connected to a 55-in 4K LG B6 OLED display with 8 million pixels of resolution that was manufactured in 2016 (LG Electronics). Evaluators wore passive polarized eyeglasses during all surgical tests. For all experiments unless otherwise indicated, the camera aperture was at 30%, a light pipe at 34% was used for target illumination (Constellation; Alcon Laboratories), and the white balance target provided by the manufacturer was used under the ideal conditions as recommended by the manufacturer.

### Ideal White Balance Technique

The following white balance technique was used for all experiments unless otherwise specified. All room lights were turned off before beginning the white balance. The white balance target, provided by the manufacturer, was placed under the operating microscope and illuminated with a 23-gauge light pipe. A 23-gauge light pipe was used during white balancing because this is the primary illumination source used during vitreoretinal surgery and is the manufacturer-recommended white balancing light source. The light pipe was directed perpendicularly at the card, and the card itself was held at a 45° angle to the optical axis of the microscope to deliver diffuse and uniform illumination to the region underneath the microscope. The card was visualized under high magnification with the image purposefully out of focus to homogenize further the uniformity of the illumination. The card was moved away from the objective lens while keeping the illumination centered on the card until the Ngenuity system indicated that we were within the brightness threshold to perform a white balance. The card then was held stationary for the entirety of the white balancing process, which took up to 5 seconds to complete.

### Photographing the ColorGauge Chart

The color target was placed in the model eye (Fig 1C) and positioned such that it filled approximately one-fifth of the frame. It is important that the target does not entirely fill the frame because this may reduce accuracy by causing significant peripheral light falloff, known as *vignetting*. Margins of at least 20% of the chart height were acquired in every image to avoid vignetting. For these analyses, it is recommended to use a light source positioned in front of the camera at a 20° to 45° angle to the target to maintain lighting uniformity and contrast while minimizing noise from target surface texture. For all experiments, the light source was a 23-gauge light pipe or 25-gauge chandelier (Alcon Laboratories) placed through a trocar and directed approximately 30° toward the target. The same trocars and model eye were used for all experiments, and additionally the light pipe was marked such that the distance of insertion through the trocar was consistent to standardize light source positioning. The target was imaged in focus such that each color patch remained distinct. Select frames from each experiment were exported in the PNG file format from the original high-definition video for further analysis (Fig 1D).

### Image Analysis and Statistical Methods

White balance and color accuracy were quantified using Imatest Master software version 5.2.5 using the Color/Tone Interactive function (Imatest), which has the capability of assessing color and tonal response, noise, signal-to-noise ratio, and dynamic range.^6^ Because the positioning of the color target can change slightly between each experiment relative to the camera frame, the region of interest was adjusted manually to ensure that the analysis area was positioned properly in each of the 30 color grids. The reference was kept at the default ColorGauge values and the standard red-green-blue color space (sRGB), where the white point is at a color temperature of 6500 K (D65 CIE illuminant standard).

The CIEDE2000 formulas are the best current color difference metric and generally are regarded as more accurate than the previous formulas.^7^ Delta E (ΔE_00_) is calculated within the Imatest software using the following equation:

$$\Delta E_{00}=\sqrt{{\left(\frac{\Delta L^{\prime }}{k_{L}S_{L}}\right)}^{2}+{\left(\frac{\Delta C^{\prime }}{k_{C}S_{C}}\right)}^{2}+{\left(\frac{\Delta H^{\prime }}{k_{H}S_{H}}\right)}^{2}+R_{T}\frac{\Delta C^{\prime }}{k_{C}S_{C}}\frac{\Delta H^{\prime }}{k_{H}S_{H}}}\;,$$

where Δ*L*ʹ is the difference in lightness from black (0) to white (100); Δ*C*ʹ is the difference in chroma; Δ*H*ʹ is the difference in hue; *k*_*L*_, *k*_*C*_, and *k*_*H*_ are parametric weighting factors set to unity; *S*_*L*_, *S*_*C*_, and *S*_*H*_ are compensations for lightness, chroma, and hue, respectively; and *R*_*T*_ is a complex mathematical transformation involving average chroma and hue. Delta C (ΔC_00_) is similar and is determined using the following equation:

$$\Delta C_{00}=\sqrt{{\left(\frac{\Delta C^{\prime }}{k_{C}S_{C}}\right)}^{2}+{\left(\frac{\Delta H^{\prime }}{k_{H}S_{H}}\right)}^{2}+R_{T}\frac{\Delta C^{\prime }}{k_{C}S_{C}}\frac{\Delta H^{\prime }}{k_{H}S_{H}}}\;.$$

However, when compared with ΔE_00_, it does not include the (Δ*L*ʹ/*k*_*L*_*S*_*L*_)^2^ term because it omits luminance difference from the calculation. Further details are available in Sharma et al,^7^ with an example of data output shown in Figure 1E.

Statistical analysis was conducted using Microsoft Excel version 16.43 (Microsoft). Delta E_00_ and delta C_00_ statistical analysis was carried out using the data analysis package integrated into the Excel software using descriptive statistics and are expressed as means ± standard deviations. Differences were calculated using the Wilcoxon matched-pairs test with *P* < 0.05 considered statistically significant.

### Experiments

A series of experiments were performed to assess color accuracy under various conditions, with each experiment designed to address a specific clinical condition. A detailed explanation for each experiment can be found in Supplemental File S1, and example outcomes are shown in Supplemental Figure S2.

#### Experiment 1: Can You Use an Alternative White Balance Target to the Manufacturer-Provided Grey Card?

The manufacturer-provided Ngenuity card was compared with a grey card (X-Rite ColorChecker Passport Video), standard 4 × 4 surgical gauze, a sheet of white paper, and the operator’s palm, as shown in Supplemental Figure S3.

#### Experiment 2: How Robust Is White Balancing When Small Deviations in Technique Occur?

The following variations were performed and compared with the standard technique: (1) holding the target card perpendicular to the optical axis of the microscope; (2) visualizing the card under low magnification; (3) placing the card in focus of the camera; (4) having the card move in and out of focus during the white balancing process; and (5) increasing ambient illumination by having the room lights on (approximately 100 lux).

#### Experiment 3: Does the Color Accuracy Drift with Time?

Color performance after white balance on day 0 was compared with that on day 180 on a machine with regular surgical use.

#### Experiment 4: Does an Active versus Passive Laser Filter Influence Color Accuracy?

The following variations were performed: (1) the laser filter remained in place for both white balancing and image acquisition; (2) the laser filter was removed before white balance and remained off for image acquisition; and (3) the laser filter was removed before white balance and then placed for image acquisition.

#### Experiment 5: If the Camera Aperture Is Adjusted during a Case, Should a Subsequent White Balance Be Performed?

White balance was performed with the aperture at 30%, with the aperture then changed to either 30%, 50%, 75%, or 100% during image acquisition, and color accuracy was determined at each setting.

#### Experiment 6: Is Color Accuracy Altered by Changing Illumination Intensity or Light Source?

White balance with a light pipe at 34% was followed by image acquisition at 34%, 20%, and 10%. The light source then was changed to a 25-gauge chandelier with further image acquisition under 25% and 50% illumination.

#### Experiment 7: Does Changing the Imaging Mode after White Balancing Change Color Accuracy?

White balance was performed under standard methods, and during image acquisition, the imaging mode was cycled between Posterior, AFX, Anterior, Hemorrhage, and Macular modes under their default settings in software version 1.2.

#### Experiment 8: Does Color Accuracy Vary between Ngenuity Machines with Differing Total Hours of Use in Different Operating Rooms?

The 3 machines were identical apart from total operating times, with machines 1, 2, and 3 having respective operational times of approximately 2760 hours (115 weeks for 3 operating days weekly), 392 hours (98 weeks for 2 operating days each month), and 196 hours (98 weeks for 1 operating day each month).

#### Experiment 9: Is Burn-in an Issue with the Organic Light-Emitting Diode Display Used in the Ngenuity System?

We assessed display uniformity and maximum luminescence using professional-grade SpyderX Elite instrument and software (Datacolor) on each system before and after multiple pixel refresh cycles. Pixel refresh is a feature of OLED televisions that can be enabled through the device menu to run automatically when users turn off the display as long as it is connected to a power source. This detects pixel deterioration and thin-film transistor changes through comparison to a set reference value and compensates as needed.

## Results

**Figure 2:**
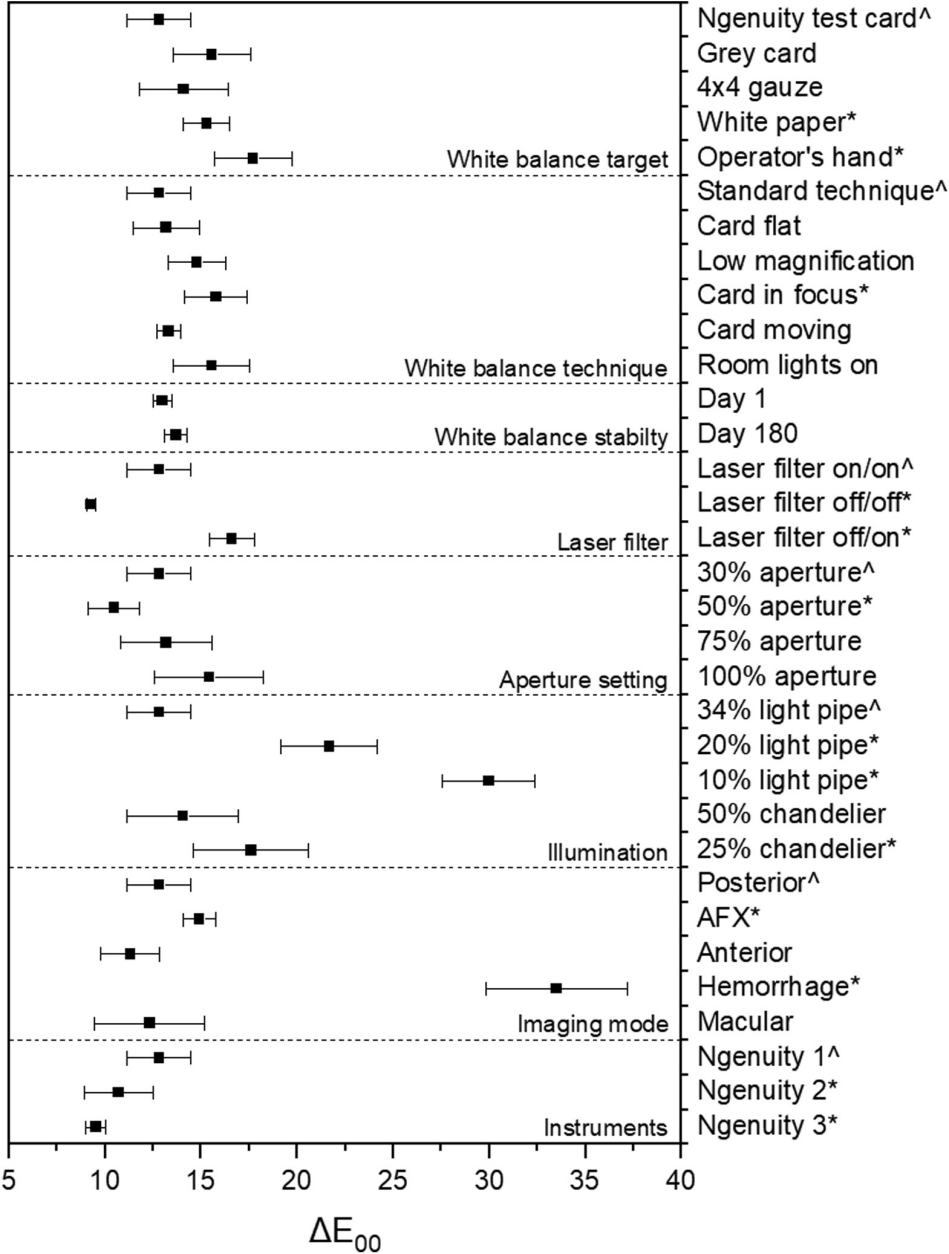
Graph showing the delta E mean and standard deviation of experiments 1 through 9, where ˆ represents the standard technique or methodology and ∗ represents those experiments that significantly differed at the 95% confidence level. AFX = air fluid exchange.

**Figure 3:**
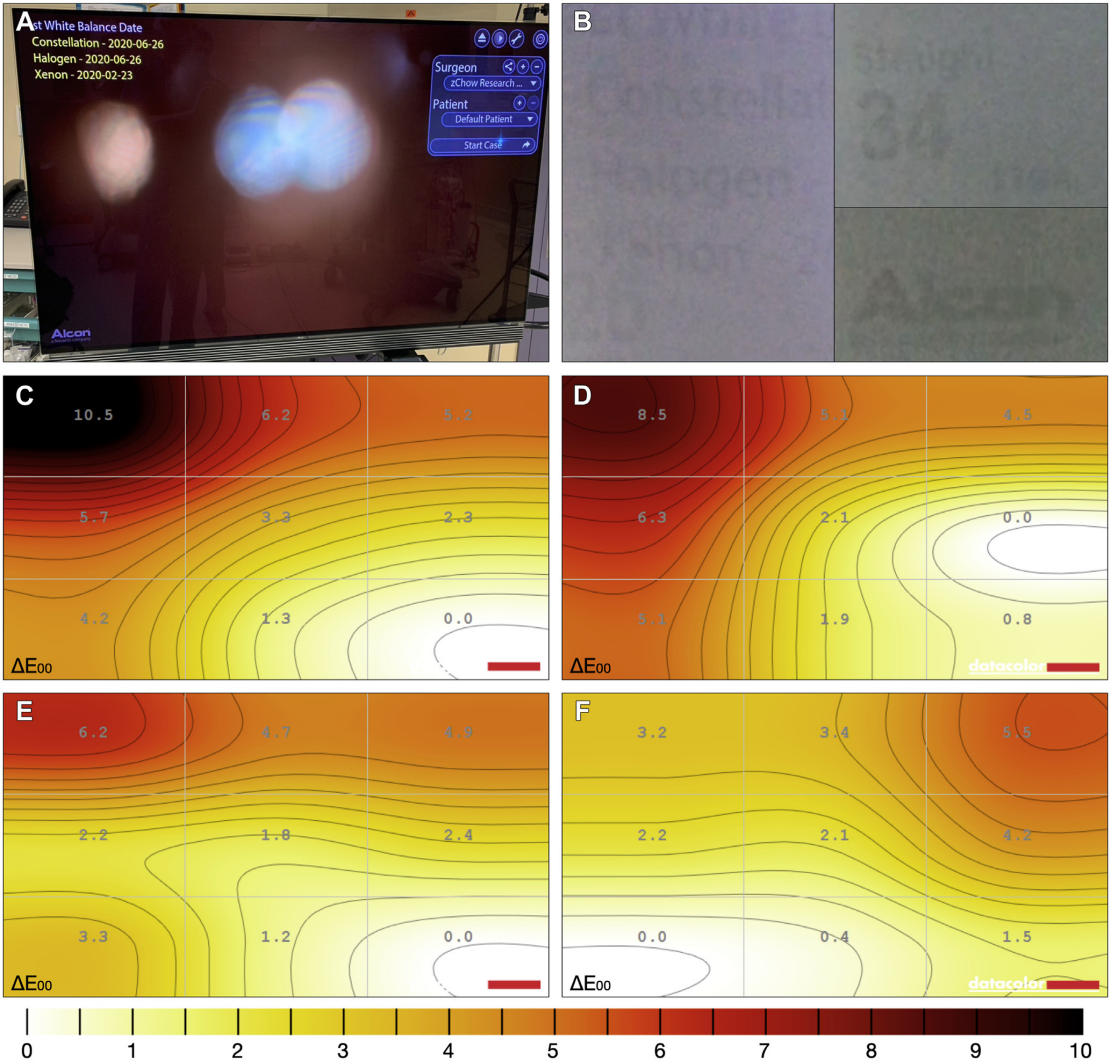
Analysis of display uniformity: (**A**) example of the static display overlays on the monitor; (**B**) examples of image retention; (**C**) display uniformity for Ngenuity system 1 as measured by delta E across 9 quadrants; (**D**) display uniformity for Ngenuity system 1 after manual pixel refresh; (**E**) display uniformity for Ngenuity system 2; and (**F**) display uniformity for Ngenuity system 3.

Color performance data for experiments 1 through 8 assessing the HDR camera can be found in Figure 2 and Table 2. Representative examples of color targets and resultant delta E plots can be found in Supplemental Figure S2 for all experiments. Display burn-in data for experiment 9 is shown in Figure 3. A further summary is provided in Supplemental Table S1.

**Table 2:** Summary of Delta E and Delta C Values for Experiments 1 through 9

Category	Method	ΔE_00_	*P* Value	ΔC_00_	*P* Value
White balance target	Ngenuity test card	12.81 ± 1.67	—	6.17 ± 0.24	—
	Grey card	15.57 ± 2.04	0.066	6.21 ± 0.21	0.785
	4 × 4 gauze	14.08 ± 2.31	0.373	6.14 ± 0.15	0.789
	White paper	15.28 ± 1.22	0.011	6.69 ± 0.10	<0.001
	Operator’s hand	17.71 ± 2.03	0.009	11.33 ± 0.92	0.002
White balance technique	Standard technique	12.81 ± 1.67	—	6.17 ± 0.24	—
	Card flat	13.18 ± 1.70	0.734	6.65 ± 0.45	0.114
	Low magnification	14.77 ± 1.50	0.079	6.76 ± 0.18	0.001
	Card in focus	15.78 ± 1.63	0.026	7.05 ± 0.09	<0.001
	Card moving	13.29 ± 0.61	0.492	6.39 ± 0.06	0.036
	Room lights on	15.53 ± 2.01	0.067	7.00 ± 0.19	<0.001
White balance stability	Day 0	12.97 ± 0.49	—	5.34 ± 0.07	—
	Day 180	13.69 ± 0.59	0.176	9.02 ± 0.04	<0.001
Laser filter	Laser filter on/on	12.81 ± 1.67	—	6.17 ± 0.24	—
	Laser filter off/off	9.28 ± 0.25	<0.001	5.18 ± 0.25	<0.001
	Laser filter off/on	16.59 ± 1.17	0.002	9.11 ± 0.56	<0.001
Aperture	30%	12.81 ± 1.67	—	6.17 ± 0.24	—
	50%	10.46 ± 1.33	0.029	5.78 ± 0.28	0.064
	75%	13.18 ± 2.35	0.790	5.87 ± 0.25	0.089
	100%	15.42 ± 2.83	0.165	5.79 ± 0.25	0.043
Illumination	34% light pipe	12.81 ± 1.67	—	6.17 ± 0.24	—
	20% light pipe	21.67 ± 2.50	0.003	8.06 ± 0.06	<0.001
	10% light pipe	29.97 ± 2.40	<0.001	9.18 ± 0.20	<0.001
	50% chandelier	14.06 ± 2.90	0.367	7.02 ± 0.27	0.003
	25% chandelier	17.60 ± 3.00	0.041	7.77 ± 0.21	<0.001
Imaging mode	Posterior	12.81 ± 1.67	—	6.17 ± 0.24	—
	AFX	14.91 ± 0.86	0.016	6.37 ± 0.19	0.167
	Anterior	11.31 ± 1.53	0.164	6.09 ± 0.24	0.623
	Hemorrhage	33.48 ± 3.68	<0.001	17.89 ± 0.37	<0.001
	Macular	12.32 ± 2.88	0.766	6.12 ± 0.31	0.804
Instruments	Ngenuity 1	12.81 ± 1.67	—	6.17 ± 0.24	—
	Ngenuity 2	10.69 ± 1.78	0.027	5.43 ± 0.48	0.003
	Ngenuity 3	9.51 ± 0.54	<0.001	5.12 ± 0.28	<0.001

– = no P-value calculated.

### Experiment 1: Can You Use an Alternative White Balance Target to the Manufacturer-Provided Grey Card?

A professional grey card (*P* = 0.07) and 4 × 4 gauze (*P* = 0.37) provided similar performance to the standard white balance target, while using white computer paper or the operator’s palm significantly increased the delta E from 12.81 ± 1.67 to 15.28 ± 1.22 (*P* = 0.01) and 17.71 ± 2.03 (*P* < 0.01), respectively.

### Experiment 2: How Robust Is White Balancing When There Are Small Deviations in Technique?

Above we outlined the standard white balance technique, and experiment 2 adjusted these steps one by one to assess the effect of various changes on color performance. The process was robust, with no significant difference in color accuracy when card position was flat instead of at 45°, magnification was low instead of high, the card was moving instead of stationary, or room lights were on instead of off. Only a small difference was found when the card was in crisp focus instead of out of focus during the white balance (15.78 ± 1.63; *P* = 0.03).

### Experiment 3: Does the Color Accuracy Drift with Time?

No significant difference or drift in color performance were found after a white balance was performed 180 days before (*P* = 0.18), demonstrating consistency over time with regular use.

### Experiment 4: Does an Active versus Passive Laser Filter Influence Color Accuracy?

Following the standard protocol with the laser filter remaining in place for both white balancing and image acquisition, a delta E of 12.81 ± 1.67 was obtained. When the laser filter was removed before white balance and remained off for image acquisition, the color performance deteriorated with a delta E of 16.59 ± 1.17 (*P* < 0.01). However, when a light filter was not engaged before white balancing, but then placed for image acquisition, color accuracy improved to 9.28 ± 0.25 (*P* < 0.01).

### Experiment 5: If the Camera Aperture Is Adjusted during a Case, Should a Subsequent White Balance Be Performed?

Increasing the aperture from 30% to 50%, which simulated a surgeon performing phacovitrectomy, improved the color accuracy to a degree that would be perceptible only under close observation (*P* = 0.03), while increasing the aperture further to 75% and 100% was not significantly different (*P* = 0.79 and *P* = 0.16, respectively).

### Experiment 6: Is Color Accuracy Altered by Changing Illumination Intensity or Light Source?

No significant difference was found when switching from a 23-gauge light pipe at 34% to a 25-gauge chandelier at 50% (*P* = 0.37). However, for both light sources with decreasing illumination, both delta E and C increased significantly, where delta E values for the light pipe at 20% and 10% were 21.67 ± 2.50 (*P* < 0.01) and 29.97 ± 2.40 (*P* < 0.01), respectively, and the delta E value for the chandelier at 25% was 17.60 ± 3.00 (*P* = 0.04).

### Experiment 7: Does Changing the Imaging Mode after White Balancing Change Color Accuracy?

No difference in color performance was found between Posterior, Macular, and Anterior modes. However, a significant difference was found between Posterior and AFX (*P* = 0.02) or Hemorrhage (*P* < 0.01) modes, with the Hemorrhage mode having a large difference in delta E because of a relative green cast of the image when this setting was engaged in the absence of blood.

### Experiment 8: Does Color Accuracy Vary between Ngenuity Machines with Differing Total Hours of Use in Different Operating Rooms?

Machine 1 versus 2 and machine 1 versus 3 showed statistically significant differences that would produce differences in color only with close observation. Machine 2 versus 3 performed similarly with no statistically significant changes.

### Experiment 9: Is Burn-in an Issue with the Organic Light-Emitting Diode Display Used in the Ngenuity System?

We found image burn-in on the machine with the heaviest use (machine 1) that was more significant with increasing brightness. After multiple attempts at manually refreshing the pixels, no improvement in the display uniformity or image retention was achieved. Finally, the brightness of the display was found to decrease with increased use, where the maximum brightness of machines 1, 2, and 3 were 386.8 cd/m^2^, 403.9 cd/m^2^, and 411.4 cd/m^2^, respectively.

## Discussion

Digitally assisted vitreoretinal surgery increasingly has been adopted worldwide for advances made possible by its HDR camera and high-resolution OLED screens. Herein, we assess its color performance and assess for the possibility of permanent image retention.

The manufacturer recommends using the provided grey card for white balancing. However, if this is misplaced, the surgeon may look for an alternative target. We tested both commercial targets and those found more conveniently in the operating room. We found that the manufacturer-provided grey card showed similar performance as a white balance target to commercially available grey cards used in the film industry. Performance also remained similar when compared with other commonly found targets that would be available in an operating room, such as gauze. However, color performance significantly deteriorated when using white computer paper or the operator’s palm because these targets are neither white nor grey. We recommend surgeons use the manufacturer-provided grey card if possible. If the manufacturer-provided grey card is misplaced, then sterile gauze can be substituted for similar color performance. This allows for an acceptable alternative sterile white-balancing technique if color performance issues are encountered intraoperatively. We recommend that objects that have a color other than grey or white should never be used as a substitute.

The manufacturer-recommended white balance technique involves numerous steps that are outlined above. We assessed 6 possible deviations from this protocol and found that only having the target in crisp focus rather than out of focus resulted in a significant change; this amounted to a delta E difference that would be perceptible only with close observation. Thus, we can reassure surgeons that although the recommended white balance process may appear detailed or complicated, minor deviations in the white balance technique often result in minimal deviations to color performance.

No set manufacturer-recommended interval to repeat white balancing exists, and surgeons may wonder how often they should perform a white balance. Therefore, we set out to assess the stability of the white balance over time. We initially attempted to assess this more frequently, but because of the coronavirus disease 2019 pandemic, we did not have access to the operating room after our initial white balance. Instead, we reassessed the color performance after 180 days on a machine that saw regular use and found no significant difference in color performance. As a result, it seems that frequent white balancing of a system is not necessary for consistent color performance. However, as mentioned already, we can show consistency in performance only up to 6 months of regular use.

Phacovitrectomy is performed in many countries, and during the procedure, it is recommended that the aperture be adjusted from 30% to 50% to reduce the depth of field for cataract extraction, followed by decreasing the aperture to 30% for the posterior segment work. The surgeon may wonder if additional white balancing is needed or if color accuracy may suffer. Color accuracy improved slightly when increasing the aperture from 30% to 50%; however, this difference was perceptible only under close observation. We recommend that surgeons performing phacovitrectomy adjust the aperture during these procedures without concern for color performance or having to perform another white balance.

Surgeons may want to switch from a light pipe to a chandelier illumination during a procedure to facilitate bimanual surgery. We found no difference in color performance at the normal illumination settings of 34% for light pipe and 50% chandelier. We recommend that surgeons using a chandelier in a particular procedure can proceed without performing another white balance during surgery. As expected, the color performance decreased when illumination decreased, and this was true for delta C, which does not include luminance difference in its calculation. Digital cameras have made dramatic improvements in performance under low-light settings, but limitations remain that are inherent to the amount of light that can reach the camera sensor. We expect this to improve in subsequent generations of the machine. We recommend that surgeons who prefer to work with lower illumination be aware of decreasing color performance and not hesitate to increase illumination until adequate.

Surgeons may use either a passive filter that remains in place for the duration of surgery or a passive filter that engages during photocoagulation. We wanted to evaluate any differences in color performance between these 2 configurations. As expected, the introduction of a 532-nm laser filter decreased color performance, but this was minimized with the use of a passive filter that remained on for both white balance and image analysis. When a laser filter was introduced after the white balance process, such as with an active laser filter, a significant decrease in color performance was noted, from 12.81 ± 1.67 to 16.59 ± 1.17. We recommend that surgeons use a passive filter or perform white balance with the laser filter engaged.

We are fortunate at our institution to have multiple Ngenuity machines at multiple surgical sites, and thus wanted to assess if consistency existed between the machines at different sites. We found no significant difference when 2 machines (machines 2 and 3) at 1 site were assessed in the same room and using the same scope. However, a small delta E difference was found that was statistically significant between machine 1 and 2 and between machine 1 and 3, respectively. This amounts to color differences that would be perceptible only under close observation and are likely the result of small optical differences in the scope and camera, as well as small differences in background illumination.

A known drawback to OLED displays is that they are susceptible to image burn-in. We sought to assess the uniformity of the display at varying brightness levels and found permanent burn-in in the machine that was used most heavily. We recommend that the Ngenuity machine be stored plugged in (but it does not need to be turned on) so that pixel refresh can be performed automatically while the machine is idle overnight. If this is not possible, then we recommend a manual pixel refresh every 2 months given that machine 2 had 49 days of use without a pixel refresh and demonstrated no signs of permanent image retention. This should help to increase the lifespan of the display. Even in machine 1, the center of the display showed similar performance to the other 2 machines, and thus burn-in did not affect the clinical use of this machine. This is likely because the center of the monitor corresponds to where surgery would be visualized, and therefore often is a dynamic image that is less susceptible to permanent image retention. Burn-in was seen on the sides of the display where static graphics are displayed for long periods. The OLED display used in our Ngenuity systems was the LG B6 from 2016. Later models of this display largely have addressed this issue, with the LG B7 manufactured in 2017 reducing image retention, or burn-in, by 50% when compared with the B6.^8^ The LG B8 of 2018 further reduced susceptibility of burn-in to less than 1% of that of the B6.^9^

Our methodology has some limitations. We chose to perform all our experiments in an operating room setting to assess real-world performance of the machine. Additionally, we limited all our experiments to air because waterproof miniaturized reference color checkers currently are not available commercially, and thus we were not able to assess the effect of balanced salt solution fluid, silicone oil, heavy liquids, or multiple interfaces on color performance.

In conclusion, we found that white balance was surprisingly robust, with few differences of clinical relevance except for use of the laser filter, illumination setting, and imaging mode. Additionally, we found permanent image retention, or burn-in, in the machine with heavy use. For every issue we identified, we offer practical advice for the surgeon to help mitigate the issue. We hope that this not only will make the surgeon aware of these potential pitfalls, but also will empower them to solve them.
