# Supplementary material for: Optimizing Color Performance of the Ngenuity 3-Dimensional Visualization System
Source: Ophthalmol Sci. 2021 Aug 24;1(3):100054. doi: 10.1016/j.xops.2021.100054 (PMC9559094; doi:10.1016/j.xops.2021.100054)
Supplement: Supplemental Figure S1 [file mmc1.pdf]

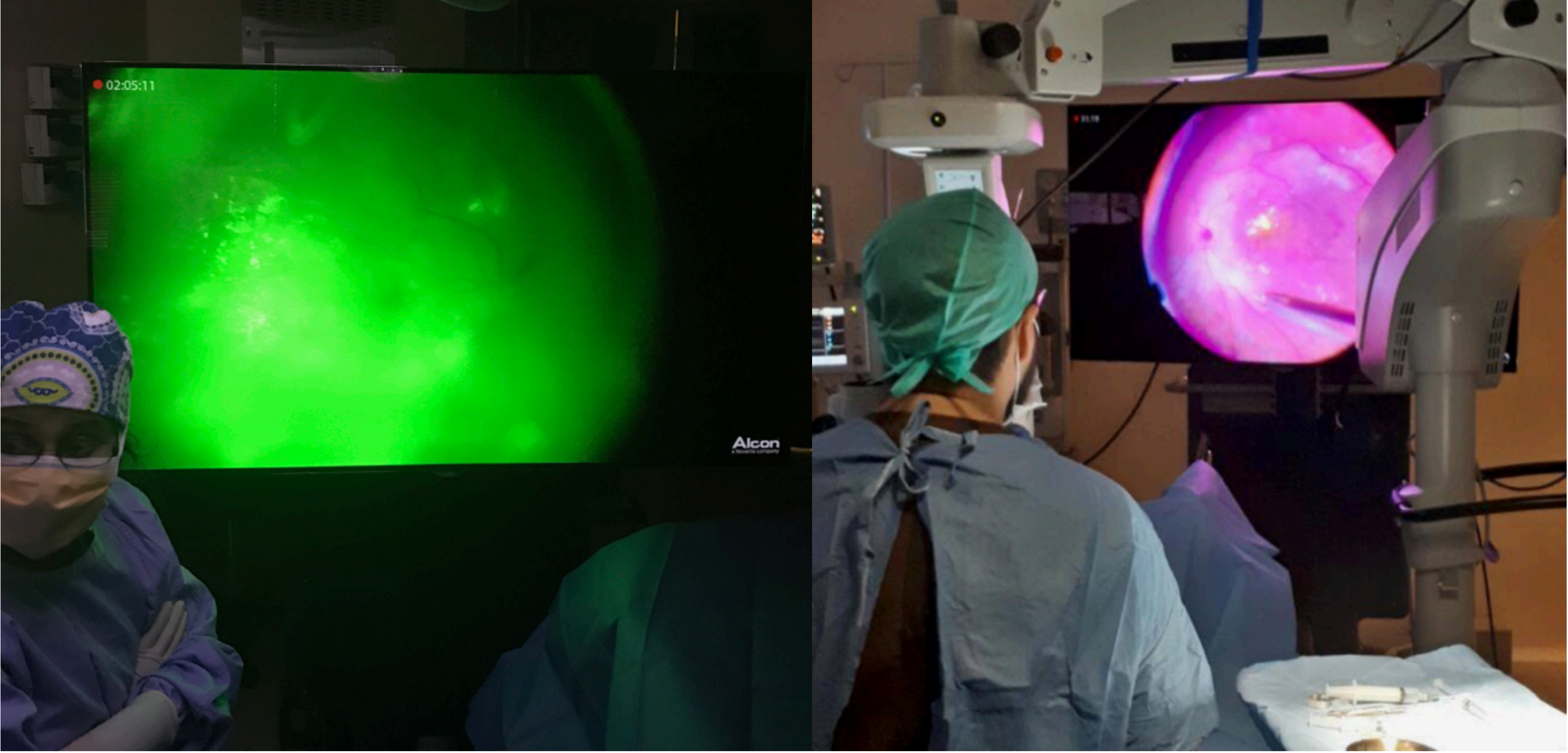

Fig. S1. Real-world examples of poor and variable color performance while using the Ngenuity® 3D Visualization System.
