# Supplementary material for: Optimizing Color Performance of the Ngenuity 3-Dimensional Visualization System
Source: Ophthalmol Sci. 2021 Aug 24;1(3):100054. doi: 10.1016/j.xops.2021.100054 (PMC9559094; doi:10.1016/j.xops.2021.100054)
Supplement: Supplemental Figure S3 [file mmc3.pdf]

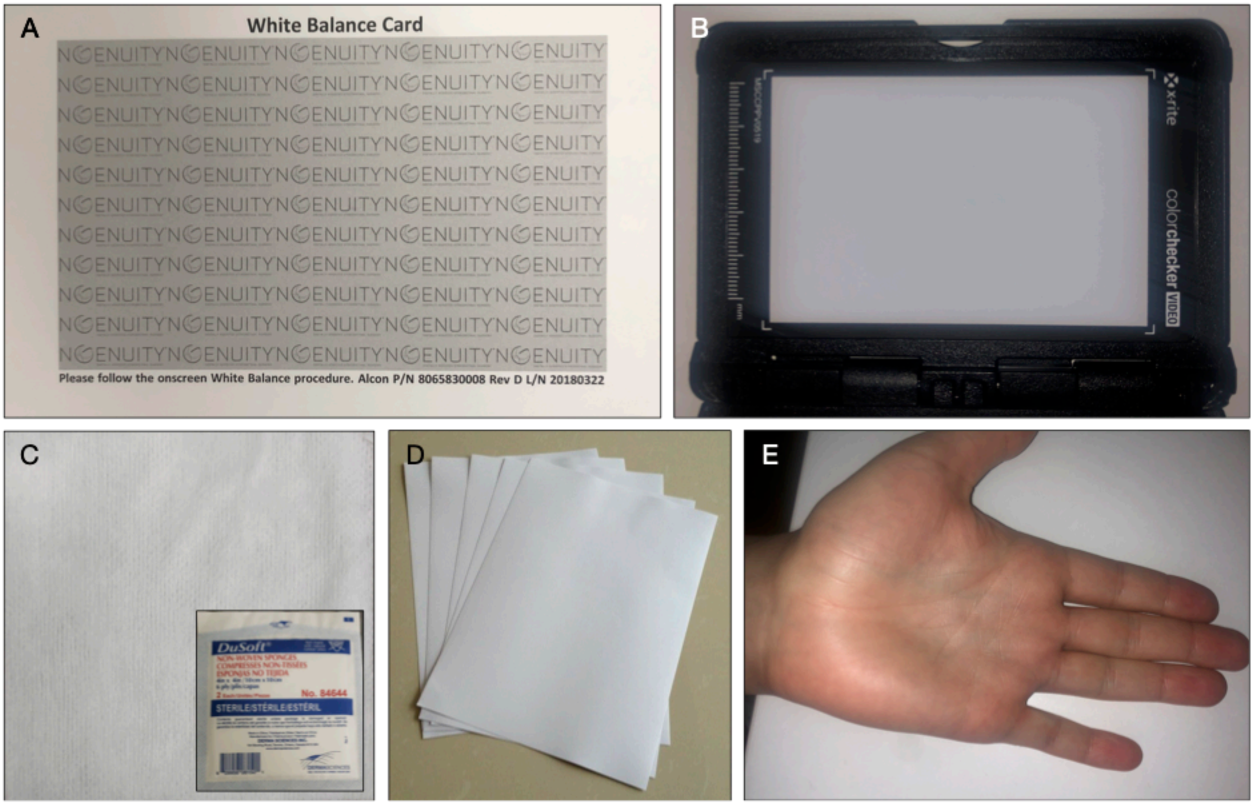

Fig. S3. White balance targets of (a) Ngenuity test card; (b) grey card; (c) 4x4 gauze; (d) standard white computer paper; and (e) the operator's palm.
