# Supplementary material for: Optimizing Color Performance of the Ngenuity 3-Dimensional Visualization System
Source: Ophthalmol Sci. 2021 Aug 24;1(3):100054. doi: 10.1016/j.xops.2021.100054 (PMC9559094; doi:10.1016/j.xops.2021.100054)
Supplement: Supplemental Table S1 [file mmc4.docx]

**Table S1.** Summary of experimental design and results.

| **Experiment** | **Question** | **Study Design** | **Findings** | **Comment** |
| --- | --- | --- | --- | --- |
| 1 | Can you use an alternative white balance target to the manufacturer-provided grey card? | The manufacturer-provided Ngenuity® card was compared to a grey card (X-Rite ColorChecker® Passport Video), standard 4x4 surgical gauze, a sheet of white paper, and the operator’s palm. | A grey card (p=0.07) and 4x4 gauze (p=0.37) provided similar performance to Ngenuity® card while white computer paper (p=0.01) and the operator's palm (p<0.01) had lower color accuracy. | We recommend using the manufacturer provided grey card if possible, but gauze can be substituted for similar color performance. |
| 2 | How robust is white balancing when there are small deviations in technique? | The standard technique was compared to the following: (1) holding the target card perpendicular to the microscopy optical axis; (2) visualizing the card under low magnification; (3) placing the card in focus of the camera; (4) having the card move in and out of focus during white balancing; and (5) increasing ambient illumination using room lighting (approximately 100 lux). | There was no significant difference in color accuracy when card was position flat instead of at 45^o^ (p=0.73), magnification was low (p=0.08), the card was moving (p=0.49), or room lights were on (p=0.07), and only a small difference was found when the card was in crisp focus (p=0.03). | Minor deviations in the white balance technique often result in minimal deviations to color performance. |
| 3 | Does the color accuracy drift with time? | Color performance following white balance on day 0 was compared with that on day 180 on a machine with regular surgical use. | There was no significant difference or drift in color performance after a white balance was performed 180 days prior (p=0.18). | Frequent white balancing is not necessary for consistent color performance, with stability up to 6 months. |
| 4 | Does an active versus passive laser filter influence color accuracy? | The (1) standard method of the laser filter remaining in place for both white balancing and image acquisition was compared with (2) removing the laser filter for both white balancing and image acquisition, and (3) white balancing without the laser filter and then placing it prior to image acquisition. | Without a laser filter for white balance and image acquisition color accuracy deteriorated (p<0.01), and when a laser filter is not engaged for white balancing but placed for image acquisition, color accuracy improved (p<0.01). | We recommend that surgeons use a passive filter or perform white balance with the laser filter engaged. |
| 5 | If the camera aperture is adjusted during a case, should a white balance be performed? | White balance was performed with the aperture at 30%, with the aperture then changed to either 30%, 50%, 75%, or 100% during image acquisition. | Increasing the aperture from 30% to 50% improved the color accuracy to a degree that would only be perceptible under close observation (p=0.03), while further increasing to 75% and 100% was not significantly different (p=0.79 and p=0.16, respectively). | Surgeons may adjust the aperture without concern for color performance or having to perform another white balance. |
| 6 | Is color accuracy altered by changing illumination intensity or light source? | White balance with a light pipe at 34% was followed by image acquisition at 34%, 20%, and 10% illumination. The light source was then changed to a 25-gauge chandelier with further image acquisition under 25% and 50% illumination. | There was no significant difference when switching from a 23G light pipe at 34% to a 25G chandelier at 50% (p=0.37). However, color accuracy suffered when decreasing the light pipe to 20% (p<0.01) and 10% (p<0.01) illumination and the chandelier to 25% illumination (p=0.04). | Surgeons who prefer to work with lower illumination should be aware of decreasing color performance and not hesitate to increase illumination until adequate. |
| 7 | Does changing the imaging mode after white balancing change color accuracy? | White balance was performed under standard methods and during image acquisition the imaging mode was cycled between Posterior, AFX, Anterior, Hemorrhage, and Macular modes under their default settings in software version 1.2. | No difference in color performance was found between Posterior and Macular (p=0.77) or Anterior (p=0.16) modes. There was decreased color accuracy under AFX (p=0.02) and Hemorrhage (p<0.01) modes. | Posterior, Macular, and Anterior imaging modes provide the greatest color accuracy. |
| 8 | Does color accuracy vary between Ngenuity® machines with differing total operational hours? | Machines 1, 2, and 3 were identical apart from having respective operational times of approximately 2760 hours, 392 hours, and 196 hours. Standard white balancing and image acquisition settings were used. | Compared to Machine 1, both Machine 2 (p=0.03) and Machine 3 (p<0.01) had improved color accuracy, although absolute differences in delta E were relatively minor. | Although statistically significant, differences in color performance would only be perceptible under close observation. |
| 9 | Is “burn-in” an issue with the OLED display used in the Ngenuity system? | We assessed display uniformity and maximum luminescence using professional grade SpyderX Elite instrument and software (Datacolor, Lucerne, Switzerland) each system before and after multiple pixel refresh cycles. | Image “burn-in” on the machine with the heaviest use (Machine 1) that was more significant with increasing brightness. There was no improvement in the display uniformity or image retention with pixel refresh. The brightness of the display decreased with increased use, where the maximum brightness of Machines 1, 2, and 3 were 386.8 cd/m^2^, 403.9 cd/m^2^, and 411.4 cd/m^2^, respectively. | We recommend that the Ngenuity® machine be stored plugged in so that pixel refresh can be automatically performed while the machine is idle overnight. If this is not possible, then we recommend a manual pixel refresh every 2 months. Image retention may not be as significant on newer OLED displays. |
