# Supplementary material for: Optimizing Color Performance of the Ngenuity 3-Dimensional Visualization System
Source: Ophthalmol Sci. 2021 Aug 24;1(3):100054. doi: 10.1016/j.xops.2021.100054 (PMC9559094; doi:10.1016/j.xops.2021.100054)
Supplement: Supplemental Figure S2 [file mmc2.pdf]

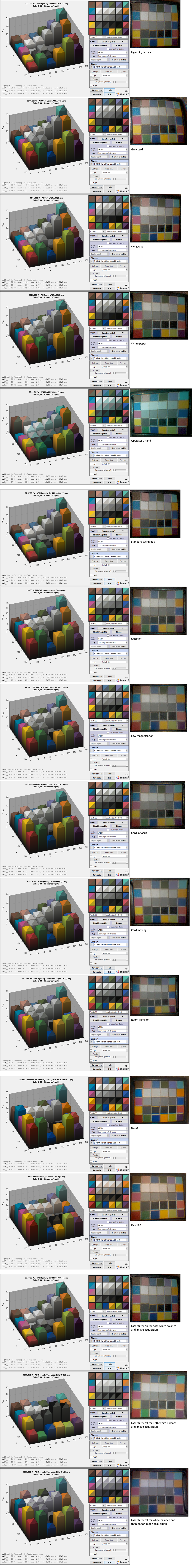

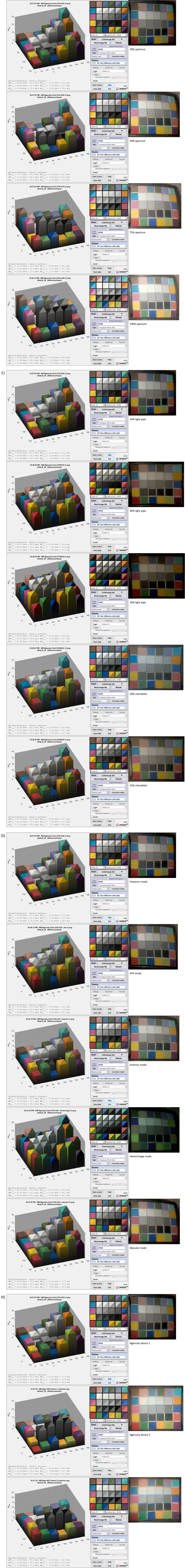

Fig. S2. Example analysis output and input image capture of the ColorGauge target for experiements 1 through 8 as described in the primary manuscript. (A) white balance target; (B) white balance technique; (C) white balance stability; (D) placement or absence of the laser filter; (E) camera aperture setting; (F) illumination source and intensity; (G) imaging mode; and (H) comparison of Ngenuity instruments.
